# PARPST: a PARallel algorithm to find peptide sequence tags

**DOI:** 10.1186/1471-2105-9-S4-S11

**Published:** 2008-04-25

**Authors:** Sara Brunetti, Elena Lodi, Elisa Mori, Maria Stella

**Affiliations:** 1 Dipartimento di Scienze Matematiche e Informatiche, Università degli studi di Siena, Siena I-53100, Italy; 2 Novartis Vaccines & Diagnostics, Siena I-53100, Italy

## Abstract

**Background:**

Protein identification is one of the most challenging problems in proteomics. Tandem mass spectrometry provides an important tool to handle the protein identification problem.

**Results:**

We developed a work-efficient parallel algorithm for the peptide sequence tag problem. The algorithm runs on the concurrent-read, exclusive-write PRAM in *O(n)* time using *log n* processors, where *n* is the number of mass peaks in the spectrum. The algorithm is able to find all the sequence tags having score greater than a parameter or all the sequence tags of maximum length. Our tests on 1507 spectra in the Open Proteomics Database shown that our algorithm is efficient and effective since achieves comparable results to other methods.

**Conclusions:**

The proposed algorithm can be used to speed up the database searching or to identify post-translational modifications, comparing the homology of the sequence tags found with the sequences in the biological database.

## Background

**Figure 1:**
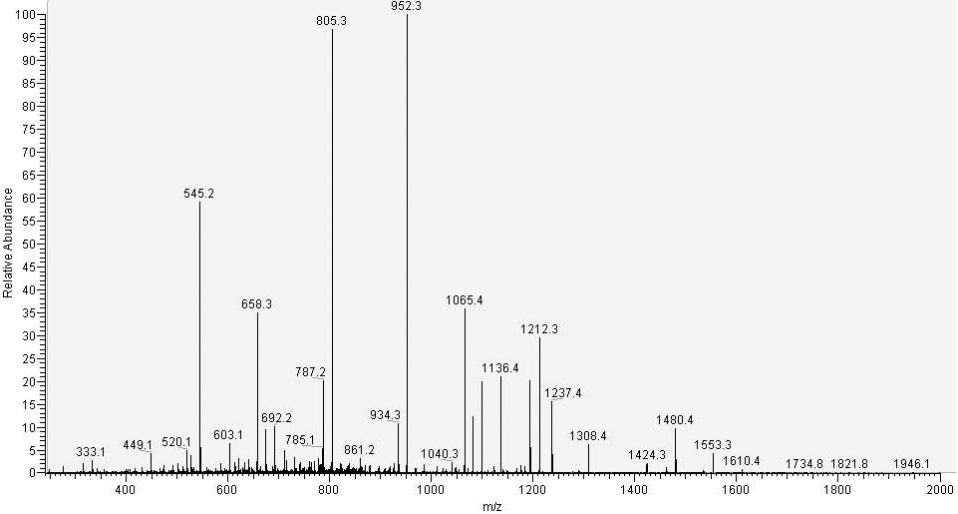
**A ms/ms spectrum**. In the *x*-axe we have the mass/charge ratio of each fragment and in the *y*-axe the abundance of it.

**Figure 2:**
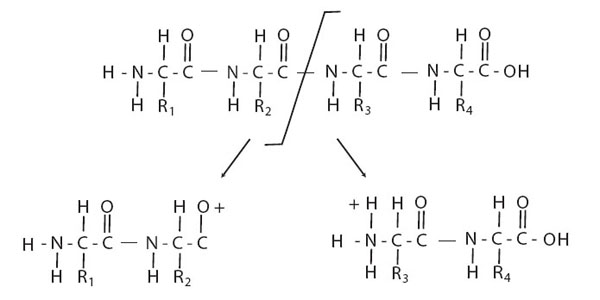
**A peptide fragmentation**. In a mass spectrometer a whole peptide (below) is broken into two fragments, one of them charged. Usually the breaking point is the peptide bond: in this case we could obtain a *b*-ion that maintains the amino termini or a *y*-ion that maintains the carboxyl termini.

Protein identification is one of the most important goals of drug discovery research and in proteomics. Nowadays, the leading technique to identify a protein or a peptide is the tandem mass spectrometry (MS/MS). The basic idea of the identification of a peptide using tandem mass spectrometry is simple: a peptide is ionized and broken, at the peptide bond, in charge fragments (ions). The mass/charge ratio of the resulted fragments are visualized in a graphic called tandem mass spectrum or ms/ms spectrum (Figure 1). A ms/ms spectrum contains: the mass of the whole peptide, and a pattern of fragments that can be associated to a given sequence. Each fragment is characterized by mass/charge ratio and intensity: we refer to this pair as peak. A good quality spectrum should contain the complete series of *y*-ions (the fragment ions containing the carboxyl terminal), and the complete series of the *b*-ions (the fragment ions containing the amino terminus) (Figure 2). In this case it is very easy to reconstruct the amino acidic sequence for the peptide, since it is sufficient to compute the mass differences between two adjacent peaks in each of the two series. Unfortunately, in practice, many factors contribute to complicate the problem. Indeed, many *b*-ion or *y*-ion peaks might be absent from the spectrum, or it might be an imperfect fragmentation that causes a different types of ions, or the sample might be contaminated, or it might be present post-translational modifications or many other type of peaks might unexpected appear in the spectrum. There are three different computational approaches motivated by peptide sequencing: the peptide identification searching in a database, the *de novo* peptide sequencing, and the peptide sequence tag. The first method finds the best matching peptide from a sequence database using a scoring function based on the likelihood that an identified peptide is actually the peptide of the spectrum [1,2]. This method is the mostly used but it is able only to identify peptide stored in a database. On the contrary the *de novo* method allows to identify a peptide using only the spectrum without any other previous knowledge and hence even if the peptide is not in a database. Many algorithms using this approach have been developed [3-10] having different time complexity, but the accuracy of them depends on the quality of the input spectrum: usually these algorithms cannot find the complete sequence due to missing peaks and hence the applicability of them is limited in practice. The peptide sequence tag approach combines the two previous methods: first the *de novo* method is applied to find a partial solution, so called sequence tag, and then a database search is applied to identify the complete sequence. The idea of using peptide sequence tags is not novel [11,12] and it is recently re-proposed to increase the speed of the database searching [13,14] or to find post-translation modifications [15]. Most of these algorithms are using a previous developed *de novo* approach and their time complexity to find the optimal solution according to any scoring function is at least *O(n^2^)*, where *n* is the number of the mass spectrum peaks. A simple scoring function can be defined as the sum of the correspondent mass peak abundances found in the spectrum [3] or it can be based on the ion-type, favouring the *b* and *y* ions over the other types [7,10]. In this paper we propose a parallel algorithm to determine all the peptide sequence tags longer than an input number of amino acids or all those scoring more than an input number, according to any scoring function. The parallel approach is motivated by demand of efficiency, since the interpretation of mass spectra is a high throughput process. The algorithm is work-efficient running in *O(n)* time on a concurrent-read, exclusive-write (CREW) PRAM [16] with *log n* processors, and it is a variation of the algorithm proposed in [5] to find peptide sequence tags. We simulate the parallelism by an implementation in Java on threads using barriers for synchronization. Our tests on 1507 spectra in the Open Proteomics Database shown that our algorithm is efficient and effective since achieves comparable results to other approaches.

## Methods

Let an experimental spectrum be given related to an unknown peptide *P* of mass *m_P_*. A peptide sequence tag is a short string of amino acid mass differences deduced from the fragment spectrum. Let any scoring function and any number δ be given. Our task is to determine all the sequence tags scoring at least δ. If the score reduces to count the length of the string, the output consists in the sequence tags of lengths at least δ. We refer to this problem as the peptide sequence tag problem.

Although a spectrum may contain a few different types of ions, for simplicity, we consider *b*-ions and *y*-ions only. Therefore we assume *M* = {*m*_1_, *m*_2_,…,*m*_n_} to represent a spectrum where the real numbers *m*_i_ correspond to the m/z ratios of the peaks in the spectrum augmented with the numbers 1, 19, *m_P_*−17, and *m_P_*+1 that represent the “empty” *b*-ion, the “empty” *y*-ion, the weightiest *b*-ion, and the weightiest *y*-ion, respectively. Let us denote the set of the masses of the twenty amino acids by *A*. The peptide sequence tag problem can be reformulated in terms of paths in a graph. We build a labelled directed acyclic graph *G = (V,E)* such that

■ every node ν_*i*_ is associated to a *m*_i_ ∈ *M* (1 ≤ *i* ≤ *n*);

■ (ν_*i*_, ν_*j*_) ∈ *E* if and only if (*m_j_* − *m_i_*) ∈ *A* (1 ≤ *i* <*j* ≤ *n*).

The peptide sequence tag problem consists in determining any path between two nodes in the graph *G* with score greater than δ. The introduction of any scoring function corresponds to assign weights to the edges of the graph: the score of a path is the sum of the scores of the edges on the path.

### The algorithm

The elements of *A* and *M* are stored in two sorted arrays in the shared global memory of the PRAM. We divide *M* into groups of *log n* consecutive elements, and we assign a “responsible” processor to each mass in each group so that the *i*th processor is responsible for the *i*th mass inside the group. We divide the algorithm in three procedures:

■ pre-computation procedure

■ propagation procedure

■ determination procedure.

We repeat each procedure for every group of *log n* elements, from the first one to the last one, and in reverse order. The procedures presented in this section are CREW since they require concurrent access to *A* and *M* in reading, but only exclusive access to the global memory in writing. In order to simplify the description that follows we give value one to the weight of each edge. This assumption corresponds to determine the longest feasible path for the *de novo* peptide sequencing problem or feasible paths longer than any given value as solutions for the peptide sequencing tag problem. In the next paragraph we describe the three procedures.

### Pre-computation procedure

The first procedure consists in building the graph. Considering each group of *log n* masses, we associate a node to each mass, and so the *i*th processor is responsible for the *i*th node ν_i_ in the group. Processor *i*, for each element in *A*, checks if there exists a node ν_*j*_ in *M* that differs from it to the mass of the element in *A*. In this case we put an edge between ν_*i*_ and ν_*j*_ in the graph. We store this edge in two different adjacency lists, the so called predecessor (*pred*) and successor (*succ*) list:

$$\begin{array}{l} pred_{i}\left[k\right]=j,\Leftrightarrow M\left[i\right]-M\left[j\right]=A\left[k\right],\\ succ_{i}\left[k\right]=j,\Leftrightarrow M\left[j\right]-M\left[i\right]=A\left[k\right]. \end{array}$$

Note that any node can have at most twenty predecessors or successors, or none. Since *M* is a sorted array, using a binary search algorithm to determine the predecessors and successors, the pre-computation takes *O(log n)* time for each group and hence *O(n)* totally.

### Propagation procedure

The second procedure of the algorithm permits, for each node, to compute the maximum length path passing through it. This goal is reached by iterating the search of the predecessor (successor) of every node using the pointer jumping technique [16] in every group. In order to handle the propagation, processor *i* stores and updates the current predecessor in the *start_path* pointer:

*start_path[i]* = *h* ∈ *{1, …, n*} ⇔ at least one path from ν_*i*_ to ν_*h*_ exists.

**Figure 3:**
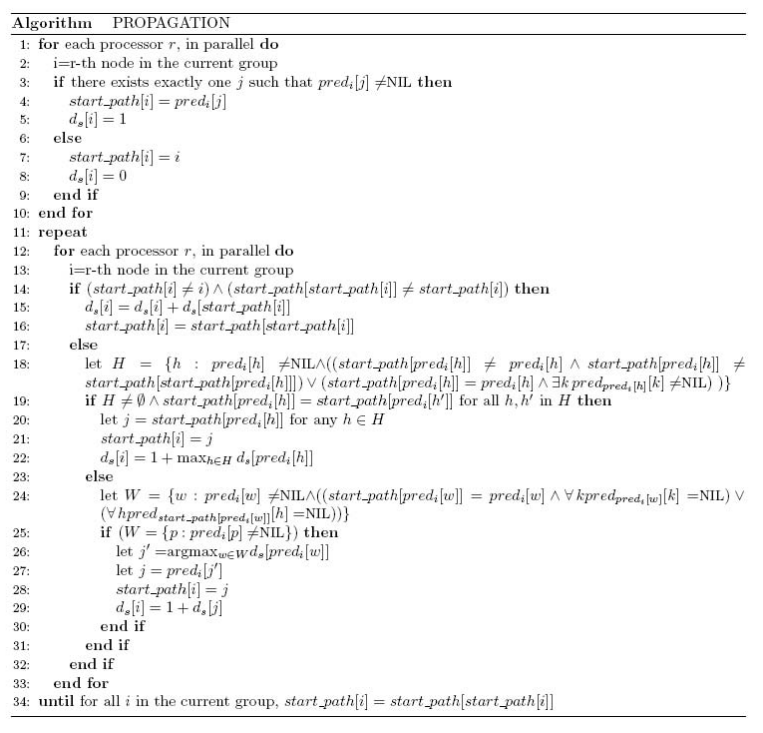
**Algorithm of the Propagation procedure**. Propagation procedure permits, for each node, to compute the maximum length path passing through it. Pseudo-code is presented for the computation of *start_path* and *d_s_* for any group, while *d _e_* and *end_path* can be obtained by replacing *pred* and *start_path* with *succ* and *end_path*, respectively.

Note that all the predecessors of ν_*i*_ belong to the ν_*i*_'s group or to any group that precedes it, being *M* sorted. Hence all the *start_path* pointers of the predecessors of ν*_i_* are known, when ν*_i_*'s group is processed, due to the order in which groups are handled. The same is done to update the current successor in *end_path*. We also calculate the distances *d_s_* and *d_e_* from any node ν*_i_* to the ν*_h_* node pointed by *start_path*[*i*] and *end_path*[*i*] pointers. At the end of the propagation procedure these two pointers, related to each node ν*_i_*, will point to the termini nodes of the longer path passing through ν*_i_*. Algorithm “Propagation” (Figure 3) describes only the computation of *d_s_* and *start_path* for any group, while *d_e_* and *end_path* can be obtained by replacing *pred* and *start_path* with *succ* and *end_path*, respectively. At first, for each node *i*, we initialize the *start_path*[*i*] pointer:

a) if *i* has only one predecessor *j*, then *start_path*[*i*] = *j* and *d_s_*[*i*] = 1 (Fig. 3, stat. 3–5);

b) otherwise, *start_path*[*i*] = *i* and *d_s_*[*i*] = 0 (Fig. 3, stat. 6–8).

**Figure 4:**
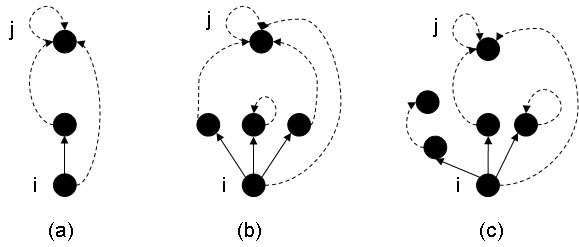
**Propagation**. Propagation procedure after the initialization, updates the *start_path* pointers. *pred* edges are represented as continued arrows, *start_path* pointers are represented as broken arrows.(a) If *start_path*[*i*] points to a node different from *i* (e.g. a predecessor of *i*) and different from *start_path*[*start_path*[*i*]] = *j*, then we assign *start_path*[*i*] = *j*. (b) If *i* has all the predecessors with *start_path* pointers pointing to themselves or predecessors with *start_path* pointing to the same node *j*, then we assign *start_path*[*i*] = *j*. (c) If all the predecessors of *i* have the *start_path* pointers cycling on themselves or *start_path* pointing to a node without predecessors, we consider the predecessor *j* having the maximum *d _s_* distance and we assign *start_path*[i]= *j*.

Then, for each node *i*, we repeat the following steps until there are no changes in any *start_path* of the group (that is *start_path*[*i*] = *start_path*[*start_path*[*i*]], for all *i* in the group):

a) if *start_path*[*i*] points to a node different from *i* and different from *start_path*[*start_path*[*i*]], then we assign *d_s_*[*i*] = *d_s_*[*i*] + *d_s_*[*start_path*[*i*]] as the new distance of the node, and *start_path*[*i*] = *start_path*[*start_path*[*i*]] for the pointer (Figure 4a; Figure 3 stat. 14–16);

b) otherwise, if *i* has all the predecessors with *start_path* pointers pointing to themselves or predecessors with *start_path* pointing to the same node *j*, then we assign *start_path*[*i*] = *j*. *d_s_*[*i*] becomes the maximum distance *d_s_* of predecessors pointing to *j*, plus 1 (Figure 4b; Figure 3, stat. 18–22);

c) otherwise, if all the predecessors of *i* have the *start_path* pointers cycling on themselves or *start_path* pointing to a node without predecessors, we consider the predecessor *j* having the maximum *d_s_* distance and we assign *start_path*[i] = *j* and *d_s_*[*i*] = 1 + *d_s_*[*j*] (Figure 4c; Figure 3, stat. 24–29);

d) otherwise, the node waits for some changes in the *start_path* pointers of its predecessors.

At the end of the propagation procedures, each node *i* knows the maximum distance *d_s_*[*i*] + *d_e_*[*i*] of a path passing through it, the starting node *start_path*[*i*], and the ending node *end_path*[*i*] of this path. The computational complexity of this procedure is *O(log n)* time for each group. Indeed, in the worst condition only one node at time is unlocked and it can upload the *start_path*. At the beginning, we have a set of pointer trees. We are interested in the sum of their heights. This sum is obviously less than *log n*. Pointer jumping and merging operations decrease the total height since a tree of height *h* is transformed into a star by applying pointer jumping in *O(log(h))* steps, and the root of a star “hooks” to the parent of any of the root's predecessor in *G*. Therefore, in the worst case, if *h_max_* is the maximum height of the initial set of, say *k*, pointer trees, all these trees degenerate into stars in *O(log(h_max_))* time, and finally they are merged in a list ranking of roots, and the algorithm stops in *O(k)* time. Since *h_max_*, *k* ≤ *log n* in every group, and we apply Algorithm “Propagation” to all the *n/log n* groups, the time complexity is *O(n)*.

### Determination procedure

This procedure allows to retrieve the solutions of the peptide sequence tag problem. Some change to the procedure permits to compute all the feasible paths of maximum length or all the feasible paths with length more than δ. We describe the latter case. At the end of the previous section, each node *i* having *d_s_*[*i*] + *d_e_*[*i*] > δ belongs to a solution of the peptide sequence tag problem. Moreover the set of the nodes *i* such that *start_path*[*i*] = *i* and *d_e_*[*i*] > δ are the starting nodes of any solution. In order to print all the solutions we can use a sequential procedure, taking at most O(*ns*), where *s* is the number of the possible sequence tags. Indeed, beginning from each starting node *i* we print all the possible solutions visiting only the successors *j* such that *d_s_*[*i*]+1+*d_e_*[*j*] > δ, and so forth for the successors of these nodes.

## Results

In order to understand the performance of our algorithm and to compare it with other existing software, we simulated the processes by using the multithreading in Java, addressing the synchronization by means of barriers. We tested our program on a four 2 GHz dual-core Intel processors 8GB RAM machine.

Our first dataset consists in 1363 annotated Escherichia Coli ion trap tandem mass spectra from the Open Proteomics Database (OPD) [17] with different *Xcorr* (97 spectra with *Xcorr* ≥ 2.5, 246 spectra with *Xcorr* ≥ 2.0 and 1363 spectra *Xcorr* ≥ 1.5), and our second dataset consists of the 280 spectra of [13]. We tested the program over all these spectra after running a data pre-processing to remove tiny noise peaks as in Mascot (personal communication).

For the first dataset, the algorithm looks for peptide sequence tags of maximum length. We evaluated the percentage of cases when the algorithm finds at least one correct sequence tag at different lengths *k*. We obtained the following percentage:

■ 99.6%, for *k* = 3;

■ 96.1%, for *k* = 4;

■ 96.1%, for *k* = 3;

■ 59.5%, for *k* = 3.

The average running time required to generate the sequence tags is 0.15 seconds. We compared our program with the public available program PepNovo on the same dataset of 280 spectra as in [13]. We evaluated the occurrence of at least one correct sequence tag in the generated sequence tag of maximum length found by our algorithm. Since in general the generated sequence tag is not unique, we used the scoring function defined in [7] to assign a score to the sequences. We evaluated the percentage of cases where any correct tag is contained in the highest scoring solution at different lengths. Additionally, we reported on the occurrence of any correct tag in the set of size three of the top scoring solutions. The results are listed in Table 1. We note that, since the percentages grow substantially if we consider the occurrence of correct sequence tags in the generated maximum length sequence tags, selectivity of the scoring function is low. Therefore better results could be obtained by using a different scoring function.

**Table 1:** Experimental results. Comparison of five tag generating methods on 280 spectra: for each tag length, algorithm and number of solution tags, the table displays the proportion of test spectra with least one correct tag.

**Tag length**	**Algorithm**	**Number of solutions**	

		**1**	**3**
**3**	Local Tag PepNovo Tag Local Tag + Guten Tag PARPST	0.529 0.804 0.725 0.493 0.761	0.764 0.925 0.855 0.732 0.839

**4**	Local Tag PepNovo Tag Local Tag + Guten Tag PARPST	0.464 0.732 0.700 0.418 0.468	0.714 0.850 0.811 0.614 0.597

**5**	Local Tag PepNovo Tag Local Tag + Guten Tag PARPST	0.410 0.664 0.571 0.318 0.236	0.593 0.764 0.696 0.464 0.407

**6**	Local Tag PepNovo Tag Local Tag + PARPST	0.332 0.579 0.527 0.079	0.489 0.632 0.546 0.125

The average running time required to generate the sequence tags is 0.11 seconds.

## Conclusions

The problem of identifying modified or variant peptide sequences is a challenging one, especially when the spectrum for unmodified sequence is not present as a standard for comparison. By joining the best partial sequences of the *de novo* interpretation and the database search algorithms, sequence tag can increase the speed and the effectiveness of the identification, and the discovery of unknown modifications, sequence variations and possibly alternate splice sites in proteins. Here, we have proposed a new work-efficient parallel algorithm to find peptide sequence tags. Our tests shown that our algorithm is efficient and accurate since achieves comparable results to other methods. Therefore, at least in theory, the proposed algorithm could be used to identify post-translational modifications, comparing the homology of the sequence tags found with the sequences in the biological database.

## List of abbreviations used

CREW – concurrent read, exclusive write

MS/MS – tandem mass (or mass/mass)

OPD – Open Proteomics Database

PRAM – Parallel Random Access Memory

RAM – Random Access Memory

## Competing interests

The authors declare that they have no competing interests.

## Authors' contributions

SB participated in the design of the algorithm, in the coordination and analysis of the tests and in revising the content of the paper. EL conceived of the initial idea to use the parallelism in this context and supervised all the phases of the developed work. EM carried out the design of the algorithm and its implementation, participated in testing the program and analysis of the experimental results and drafted the manuscript. MS carried out the testing and debugging of the program, participated in the analysis of the experimental results, and helped in revising the paper. All authors read and approved the final manuscript.

## References

[B1] Eng J, McCormack A, Yates J (1994). An approach to correlate tandem mass spectral data of peptides with amino acid sequences in a protein database. Journal of American Society of Mass Spectrometry.

[B2] Perkins D, Pappin D, Creasy D, Cottrell J (1999). Probability-based protein identification by searching sequence databases using mass spectrometry data. Electrophoresis.

[B3] Bafna V, Edwards N, Vingron M, Istrail S, Pevzner P, Waterman M (2003). On de novo interpretation of tandem mass spectra for peptide identification. Proceedings of the seventh annual international conference on Computational molecular biology: 10-30 April 2003; Berlin.

[B4] Brunetti S, Dutta D, Liberatori L, Mori E, Varrazzo D, Ribeiro B, Albrecht RF, Dobnikar A, Pearson DW, Steele NC (2005). An efficient algorithm for de novo peptide sequencing. Proceeding of the seventh international conference on Adaptive and Natural Computing Algorithms: 21-23 March 2005; Coimbra.

[B5] Brunetti S, Lodi E, Mori E, Hochreiter S, Küng J, Palkoska J, Wagner R (2007). De novo peptide sequencing: a work-efficient parallel algorithm. Proceeding of the First International Conference on Bioinformatics Research and Development: 12-14 March 2007; Berlin.

[B6] Chen T, Kao MK, Tepel M, Rush J, Church GM, Shmoys D: Society for Industrial and Applied Mathematics (2000). A dynamic programming approach to de novo peptide sequencing via tandem mass spectrometry. Proceedings of the eleventh annual ACM-SIAM symposium on Discrete algorithms: 09-11 January 2000; San Francisco.

[B7] Danĉík V, Addona TA, Clauser KR, Vath JE, Pevzner PA (1999). De novo peptide sequencing via tandem mass spectrometry. Journal of Computational Biology.

[B8] Frank A, Pevzner PA (2005). Pepnovo: de novo peptide sequencing via probabilistic network modeling. Anal Chem.

[B9] Lu B, Chen T (2003). A suboptimal algorithm for de novo peptide sequencing via tandem mass spectrometry. Journal of Computational Biology.

[B10] Ma B, Zhang K, Hendrie C, Liang C, Li M, Doherty-Kirby A, Lajoie G (2003). PEAKS: Powerful Software for Peptide De Novo Sequencing by MS/MS. Rapid Communications in Mass Spectrometry.

[B11] Mann M, Wilm M (1994). Error-tolerant identification of peptides in sequence databases by peptide sequence tags. Analytical Chemistry.

[B12] Han Y, Ma B, Zhang K (2005). Spider: software for protein identification from sequence tags with de novo sequencing error. Journal of Bioinform Comput Biol.

[B13] Frank A, Tanner S, Bafna V, Pevzner P (2005). Peptide sequence tags for fast database search in mass-spectrometry. Journal of Proteome Research.

[B14] Tabb DL, Saraf A, Yates JR (2003). Gutentag: high-throughput sequence tagging via an empirically derived fragmentation model. Analytical Chemistry.

[B15] Liu C, Yan B, Song Y, Xu Y, Cai L (2006). Peptide sequence tag-based blind identification of post-translational modifications with point process model. Bioinformatics.

[B16] Jájá J (1992). An introduction to parallel algorithms.

[B17] Prince JT, Carlson MW, Wang R, Lu P, Marcotte EM (2004). The need for a public proteomics repository. Nat Biotechnol.

